# Supplementary material for: Role of Noradrenaline and Adrenoreceptors in Regulating Prostaglandin E2 Synthesis Cascade in Inflamed Endometrium of Pigs
Source: Int J Mol Sci. 2023 Mar 20;24(6):5856. doi: 10.3390/ijms24065856 (PMC10054430; doi:10.3390/ijms24065856)
Supplement: Supplementary file 1 [file ijms-24-05856-s001.zip › ijms-2196598-supplementary.pdf]

Supplementary material

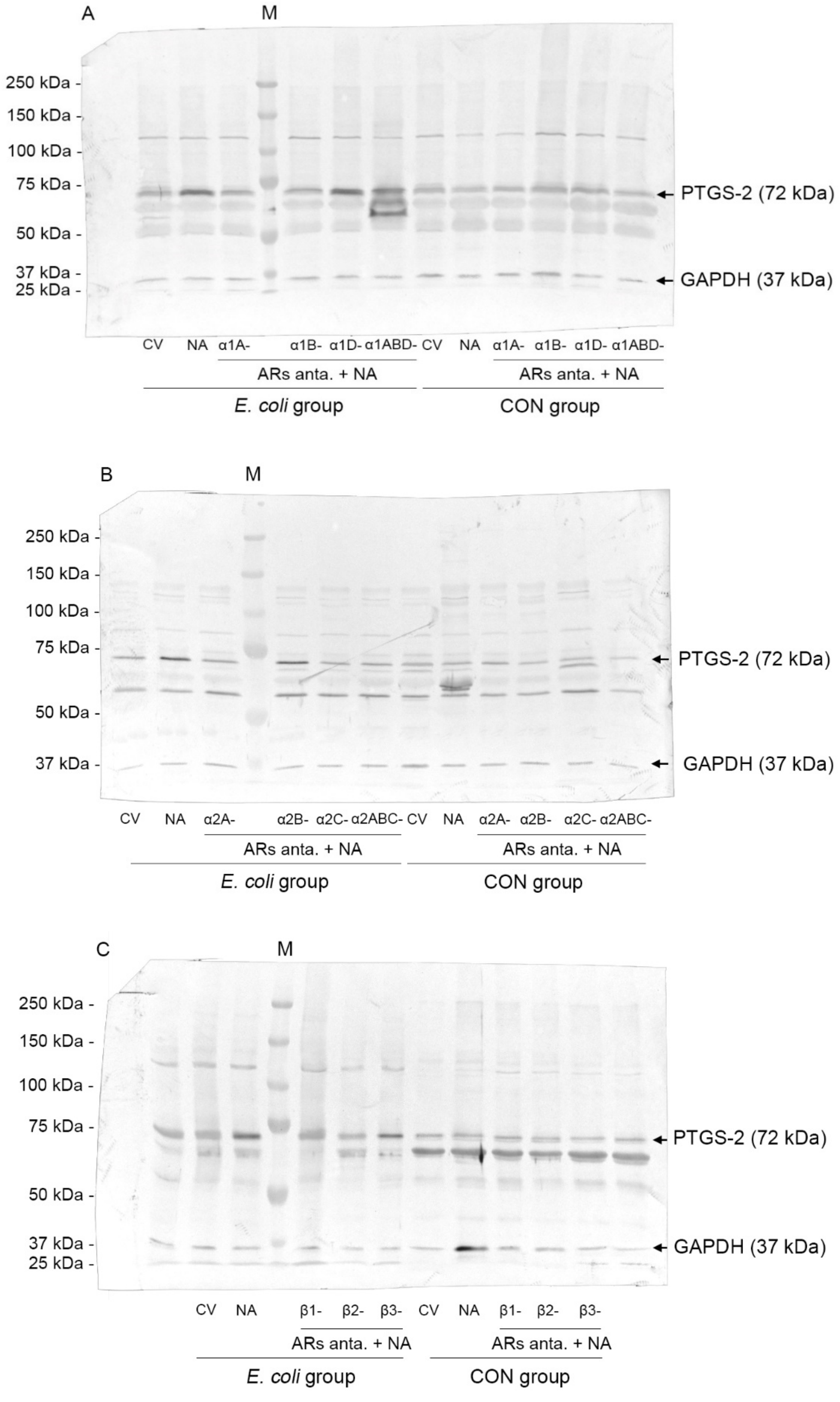

Supplementary Figure S1. Western blot analysis of prostaglandin-endoperoxidase synthase-2 (PTGS-2) in pig endometrium from the control (CON) and *E. coli* (*E. coli*) groups after using noradrenaline alone or antagonists of α1- (blot A), α2- (blot B) and β (blot C) -adrenoreceptors with

noradrenaline. The density of bands was normalized in relation to glyceraldehyde-3-phosphate dehydrogenase (GAPDH). For PTGS-2 antibody bands are visible at 72 kDa, for GAPDH antibody at 37 kDa. marker: M; control value: (CV, untreated tissue); noradrenaline: NA; adrenoreceptors: ARs; antagonist: anta.

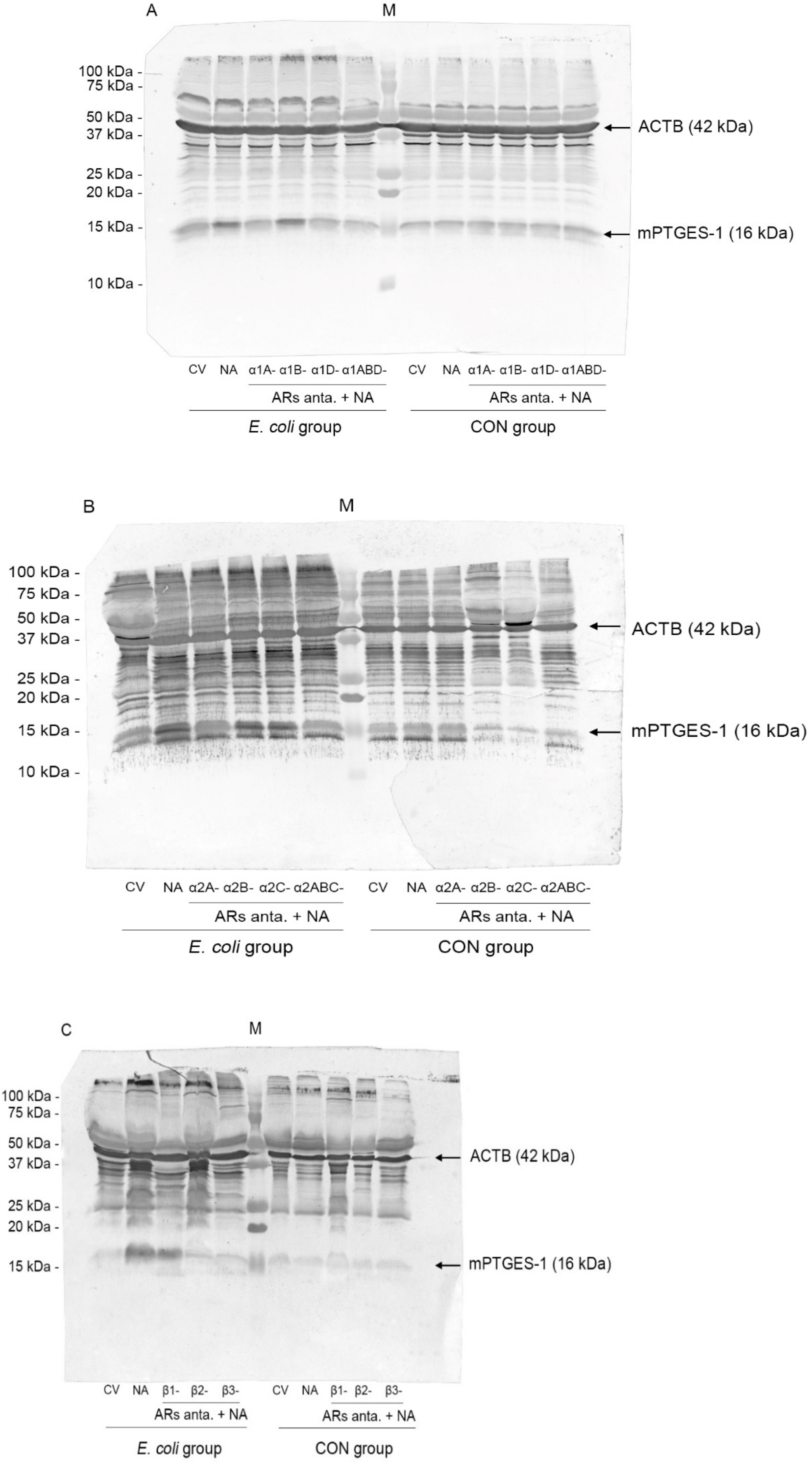
A
M
100 kDa -
75 kDa -
50 kDa -
37 kDa -
25 kDa -
20 kDa -
15 kDa -
10 kDa -
ACTB (42 kDa)
mPTGES-1 (16 kDa)
CV NA α1A- α1B- α1D- α1ABD-
ARs anta. + NA
E. coli group
CV NA α1A- α1B- α1D- α1ABD-
ARs anta. + NA
CON group
B
M
100 kDa -
75 kDa -
50 kDa -
37 kDa -
25 kDa -
20 kDa -
15 kDa -
10 kDa -
ACTB (42 kDa)
mPTGES-1 (16 kDa)
CV NA α2A- α2B- α2C- α2ABC-
ARs anta. + NA
E. coli group
CV NA α2A- α2B- α2C- α2ABC-
ARs anta. + NA
CON group
C
M
100 kDa -
75 kDa -
50 kDa -
37 kDa -
25 kDa -
20 kDa -
15 kDa -
ACTB (42 kDa)
mPTGES-1 (16 kDa)
CV NA β1- β2- β3-
ARs anta. + NA
E. coli group
CV NA β1- β2- β3-
ARs anta. + NA
CON group

Supplementary Figure S2. Western blot analysis of microsomal prostaglandin E synthase-1 (mPTGES-1) in pig endometrium from the control (CON) and *E. coli* (*E. coli*) groups after using noradrenaline alone or antagonists of α1- (blot A), α2- (blot B) and β (blot C) -adrenoreceptors with noradrenaline. The density of bands was normalized in relation to β-actin (ACTB). For mPTGES-1 antibody bands are visible at 16 kDa, for ACTB antibody at 42 kDa. marker: M; control value: (CV, untreated tissue); noradrenaline: NA; adrenoreceptors: ARs; antagonist: anta.
